# A search for protein biomarkers links olfactory signal transduction to social immunity

**DOI:** 10.1186/s12864-014-1193-6

**Published:** 2015-02-08

**Authors:** Maria Marta Guarna, Andony P Melathopoulos, Elizabeth Huxter, Immacolata Iovinella, Robert Parker, Nikolay Stoynov, Amy Tam, Kyung-Mee Moon, Queenie WT Chan, Paolo Pelosi, Rick White, Stephen F Pernal, Leonard J Foster

**Affiliations:** Department of Biochemistry & Molecular Biology, Centre for High-Throughput Biology, University of British Columbia, 2125 East Mall, Vancouver, BC V6T 1Z4 Canada; Beaverlodge Research Farm, Agriculture & Agri-Food Canada, Beaverlodge, AB T0H 0C0 Canada; Kettle Valley Queens, Grand Forks, BC Canada; Department of Agriculture, Food and Environment, University of Pisa, Pisa, Italy; Department of Statistics, University of British Columbia, Vancouver, BC V6T 1Z4 Canada; Current address: Dalhousie University, Halifax, NS Canada; Current address: Macquarie University, Sydney, NSW Australia

## Abstract

**Background:**

The Western honey bee (*Apis mellifera* L.) is a critical component of human agriculture through its pollination activities. For years, beekeepers have controlled deadly pathogens such as *Paenibacillus larvae, Nosema* spp. and *Varroa destructor* with antibiotics and pesticides but widespread chemical resistance is appearing and most beekeepers would prefer to eliminate or reduce the use of in-hive chemicals. While such treatments are likely to still be needed, an alternate management strategy is to identify and select bees with heritable traits that allow them to resist mites and diseases. Breeding such bees is difficult as the tests involved to identify disease-resistance are complicated, time-consuming, expensive and can misidentify desirable genotypes. Additionally, we do not yet fully understand the mechanisms behind social immunity. Here we have set out to discover the molecular mechanism behind hygienic behavior (HB), a trait known to confer disease resistance in bees.

**Results:**

After confirming that HB could be selectively bred for, we correlated measurements of this behavior with protein expression over a period of three years, at two geographically distinct sites, using several hundred bee colonies. By correlating the expression patterns of individual proteins with HB scores, we identified seven putative biomarkers of HB that survived stringent control for multiple hypothesis testing. Intriguingly, these proteins were all involved in semiochemical sensing (odorant binding proteins), nerve signal transmission or signal decay, indicative of the series of events required to respond to an olfactory signal from dead or diseased larvae. We then used recombinant versions of two odorant-binding proteins to identify the classes of ligands that these proteins might be helping bees detect.

**Conclusions:**

Our data suggest that neurosensory detection of odors emitted by dead or diseased larvae is the likely mechanism behind a complex and important social immunity behavior that allows bees to co-exist with pathogens.

**Electronic supplementary material:**

The online version of this article (doi:10.1186/s12864-014-1193-6) contains supplementary material, which is available to authorized users.

## Background

The health of honey bees (*Apis mellifera* L.) is crucial for honey production and pollination of a wide variety of crops. The contribution of honey bees to Canadian agriculture exceeds $2.3 billion (Alex Campbell, Agriculture & Agri-Food Canada. Personal communication) while the value added to crops such as almonds, berries, fruits, vegetables and other nuts in the U.S. is estimated to be $11.7 billion [1]. Winters, in particular, are a profound determinant of colony survival. Prior to 2006, overwintering mortality of honey bee colonies in North America was 10 to 15%; however, losses in North America and Europe have dramatically increased to an average of approximately 30% [2]. While this has not yet had a discernable effect on crop yields, it has made it much more challenging for beekeeping companies to remain solvent. The causes of honey bee losses have been attributed to a multitude of factors [3], including bee-specific pathogens and parasites such as the mite *Varroa destructor* and the microsporidia *Nosema apis* and *Nosema ceranae*. Also, long-implicated as a leading cause of colony mortality before the introduction of *V. destructor*, the bacterial brood pathogen *Paenibacillus larvae* that causes American Foulbrood continues to be a problem [4]. *V. destructor* is now considered the single greatest natural threat to honey bees worldwide, as it weakens and kills colonies by parasitizing bees as well as vectoring several viruses that may be even more virulent to bees than the mites themselves [5].

Though acaricides, antibiotics and fungicides are registered for controlling *V. destructor, P. larvae* and *Nosema* spp., a number of negative consequences are associated with their use. These include the economic cost of the treatments themselves, concerns around the potential contamination of hive products [6], widespread antibiotic [7] and acaricide resistance [8-11], and concerns over the effectiveness of chemotherapy for controlling *Nosema* spp [12]. Indeed, these pathogens are on a path akin to a chemical treadmill whereby resistance develops within a few years of the initial use of a particular chemical [13]. At the same time, viruses’ impact on bee health continues to increase and no conventional treatments are available to counter them. The phenomenon of drug resistance is not recent, even in microlivestock such as bees, and will likely become more widely spread. Spivak and Gilliam observed over ten years ago that acaricides and antibiotics were no longer effective against *Varroa* and *Paenibacillus larvae* [14].

An alternative pest management approach is to identify and select bees with an increased ability to tolerate diseases without chemical intervention. While this could be achieved through heightened innate immunity, honey bees, as eusocial animals, have the added capacity for social behaviors that enable resistance to pathogens. Several behaviors that help to confer colony-level resistance against parasites and pathogens have been characterized, including hygienic behavior (HB) [15,16], *Varroa* Sensitive Hygiene (VSH) [17], grooming behavior [18,19] and others [14]. HB is the best understood and it involves the detection of dead or diseased bees in brood cells, uncapping of cells and removal of the affected larvae or pupae by nurse bees. The primary means by which HB confers disease resistance is thought to be the continual elimination of brood pathogens from the hive environment, which would otherwise remain, multiply and potentially infect other bees. In the case of *P. larvae*, with which *A. mellifera* has had the longest time to co-evolve, bees specifically remove infected larvae or pupae when the bacteria are still in the vegetative state [20]. Although the term ‘hygienic behavior’ was originally used to describe removal of brood infected with *P. larvae*, its use has gradually been expanded to describe the removal of brood infected with chalkbrood disease (caused by *Ascosphaera apis*), as well as brood parasitized by *Varroa* [21]. It has further been applied to brood invaded by the greater wax moth, *Galleria mellonella* [22], or brood infested with the small hive beetle, *Aethina tumida* [23]. The magnitude of HB varies between individual colonies and populations, making it possible to selectively breed bees to enrich HB [20,24], with the goal of increasing the ability of the bee population to manage disease while reducing beekeeper intervention and chemical treatments. Testing colonies for selective breeding, however, is a slow and resource-intensive process that could be facilitated by more rapid molecular assays.

We have previously reported that larval proteins involved in chitin biosynthesis, wound healing and innate immunity pathways are correlated with HB and VSH [25]. In the same study, we discovered that HB and VSH were also associated with the expression of antennal proteins in functional classes such as cell surface-linked signaling and protein modification pathways. In the current study, we extend our original approach to cover three generations, two geographically distinct sites and far more colonies (N = 167) towards two goals: 1) identify prognostic biomarkers that could be used for marker-assisted selection and 2) better understand the mechanisms underlying behaviors that confer social immunity.

## Results

### Enrichment of HB by selective breeding in GF

Differences in a phenotype such as a behavior must be the result of altered protein expression or activity so our guiding hypothesis was that honey bees exhibiting hygienic and other social immunity behaviors should display unique protein expression profiles, at least in the tissues/organs involved in the behaviors. If these unique profiles could be accurately characterized then the proteins most highly up or down-regulated relative to ‘normal’ bees would make excellent biomarkers for selective breeding and they would also be likely to have a mechanistic role in the manifestation of the behavior. HB is heritable so it follows that the expression patterns of proteins involved in HB must also be heritable so we first established that we could selectively breed for HB efficiently.

**Figure 1:**
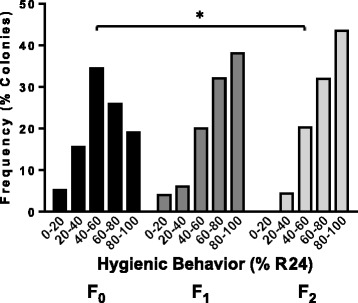
**Selective Breeding of hygienic behavior (HB).** In an initial breeding experiment, colonies were selectively bred for HB over three generations to test the efficacy of selection. For each generation, colonies expressing the highest level of HB estimated by the fraction of freeze-killed pupae removed after 24 hours (R24) were selected as breeders and their virgin queens and drones close mated in an isolated apiary. F_0_ bars represent the natural frequency distribution of HB in the starting population, while the F_1_ and F_2_ bars represent the distribution after one and two rounds of selection, respectively. The frequency distribution in the F_1_ and F_2_ is significantly different from the F_0_ (*P = .0002, one-way ANOVA).

Prior to the experiments aimed to evaluate protein expression patterns and to identify HB markers, we performed a small selective breeding program to confirm that social immunity traits, particularly HB, could be enriched in our apiaries based on field testing. Using a closed mating system and the field-based liquid nitrogen freeze-kill test [14] as a test of HB, we observed a significant enrichment of the trait over only three generations (Figure 1)(P = .0002, F = 8.719, df = 2, one-way ANOVA of frequency distribution data). The distribution of HB in the population changed dramatically in the first round of selective breeding, and the HB values continued to improve in the second round of selective breeding. Further, the proportion of colonies with high HB scores (higher than 90% cells removed after 24 h) increased from 12% in the first year to 18% the second year and to 26% in the third year.

### Wide range of hygienic behavior in starting populations

**Figure 2:**
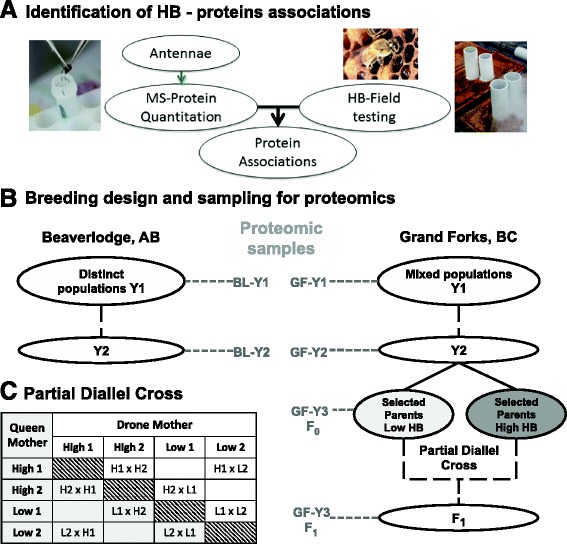
**Breeding and sampling design for the identification of heritable protein markers. A**. Diagram illustrating our approach to identify specific proteins associated with HB behavior using mass spectrometry. **B**. Breeding design and origin of samples. Parallel breeding programs were conducted in Beaverlodge, Alberta (55°N, 119°W) and Grand Forks, British Columbia (49°N, 118°W). Samples for proteomic analysis were taken each year at each location. The partial diallel cross used with the instrumental insemination in AB and in Y3 in BC is shown in **C**.

**Figure 3:**
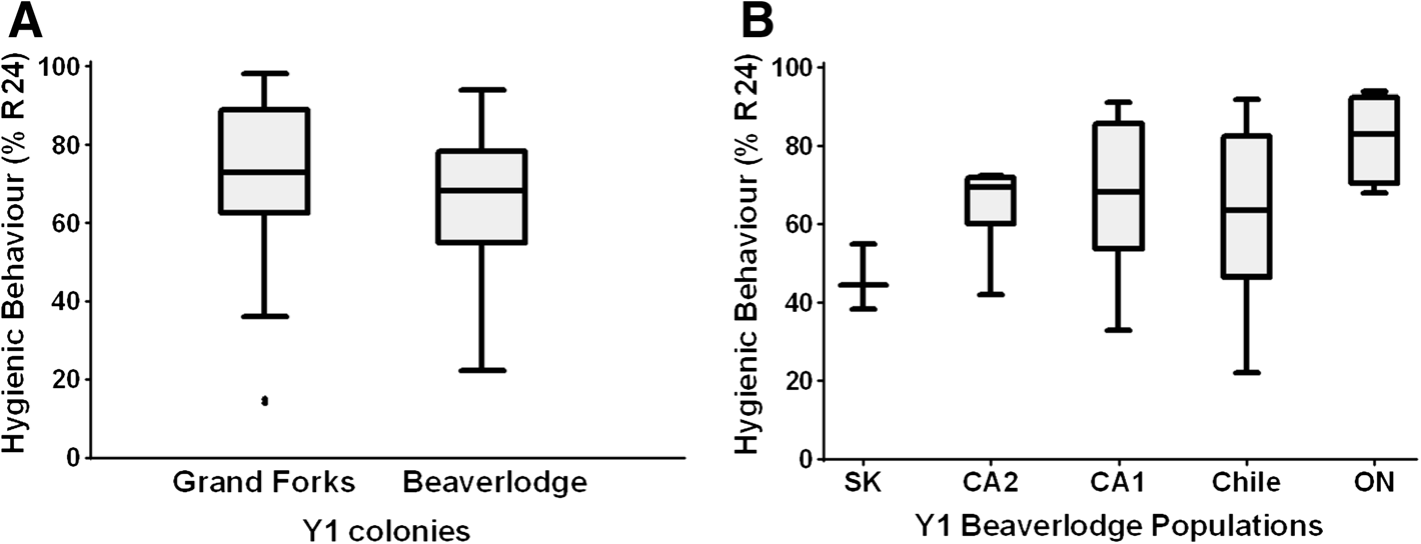
**Hygienic behavior of starting populations. A**. HB of the Y1 colonies in Grand Forks, BC and at the Research Farm in Beaverlodge, Alberta, showed a wide range of HB values as determined by the proportion of cells that were uncapped and pupae were removed after 24 h (R24h) using the liquid nitrogen freeze-killed brood method. **B**. The five populations analysed in Beaverlodge, one originated in Saskatchewan (SK), two in California (CA1 and CA2), one in Chile (Ch) and one in Ontario (ON), also showed a wide range of HB.

We then explored the correlation between protein expression and HB to identify proteins correlated with the behavior. We established two populations in geographically distinct areas of Western Canada and proceeded to breed progeny with divergent levels of HB and measured protein expression profiles for each generation (Figure 2). The starting colonies at each location presented a wide range of HB (Figure 3) and were assembled from geographically diverse sources of stock used by Canadian beekeepers. Breeding was performed as outlined in Methods using instrumental insemination of drones and virgin queens from high- and low-scoring colonies was used to achieve a controlled partial diallel crosses which created high and low scoring colonies, as well as hybrids, intended to facilitate the identification protein expression related to HB, and to estimate heritability of the protein markers (Figure 2).

**Figure 4:**
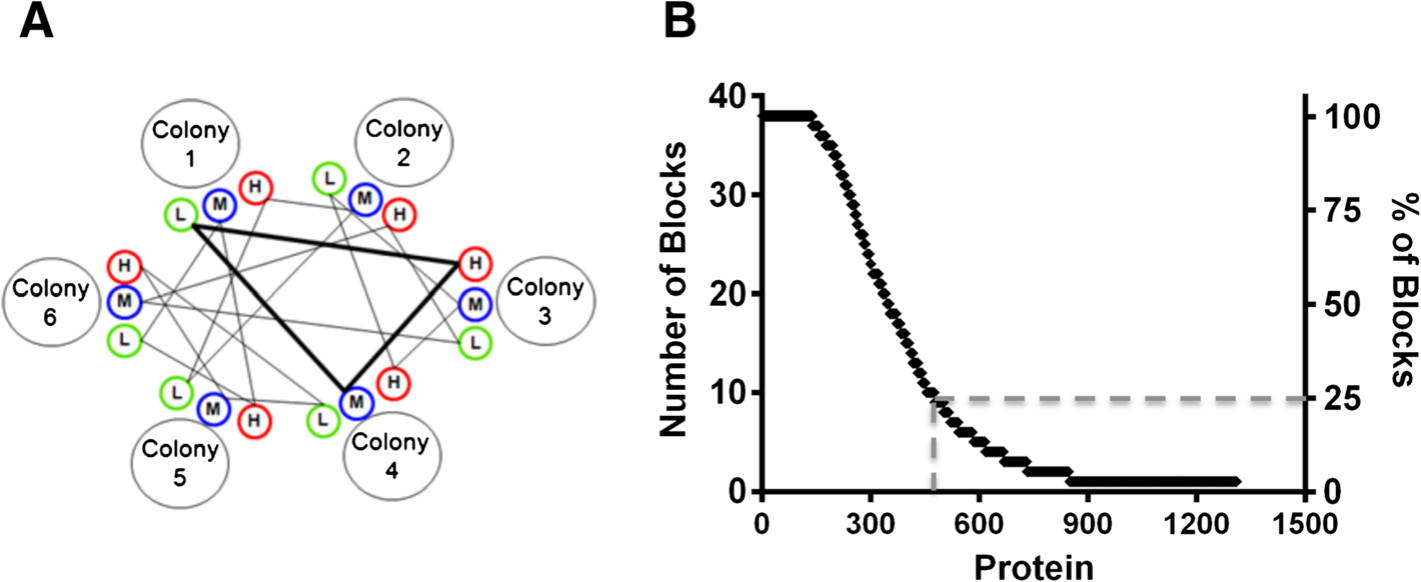
**Structure and results of the randomized incomplete block design. A**. The matrix illustrated below allowed for a comparison of the protein profiles of all colonies. Each colony was sampled in triplicate and each sample was labeled with either light, medium or heavy isotopes, and was assigned to a block. Each block, represented by a triangle, contained three samples from different colonies and labels and was analysed in one Mass Spectrometry run. **B**. Due to the semi-stochastic nature of data-dependent acquisition in LC-MS/MS and the wide range of protein abundances, not all proteins were detected in every sample. Shown is a representative plot of the frequency where 1312 proteins were detected across 38 blocks in BL-Y1 sample set. The 476 proteins that were detected in at least 10 (25%) of the blocks were considered in the analysis.

Antennae of workers on brood frames were collected from all colonies in each of the generations. The choice of tissue to focus on is obviously important when studying expression markers (as opposed to quantitative trait loci where all cells and tissues would be expected to have the same genotype) and as such we were guided by the following rationale: what causes bees to be hygienic is not known but it may involve either a heightened sensitivity to detect a specific signal or a unique wiring of the brain that causes hygienic bees to respond to a signal differently. The former possibility would likely manifest as differences in sensory organs (antennae are bees’ primary sensory organ) while the latter would mean differences in their brains. Le Conte *et al.* [26] reported the analysis of brain transcriptome of highly *Varroa*-hygienic bees and the identification of a set of genes involved in social immunity. Although the function of these candidate genes did not seem to support higher olfactory sensitivity in hygienic bees as previously hypothesized, the authors noted that the analysis of peripheral tissues, like antennae, should be performed since insect behavior can be dramatically affected by changes in expression of antennal-specific genes. We, therefore, elected to focus on the proteomic analysis of antennae. Proteins were extracted from the collected antennae and the protein expression pattern of each colony was measured by mass spectrometry, employing a triplexed stable isotope labelling approach that we have applied previously [25] in a randomized incomplete block (Figure 4a, see Methods).

Approximately 1300 proteins were quantifiable in the analyses of each generation and, of these, approximately 500 in each set were represented in at least 25% of the ‘blocks’ (triplex-labelled samples, Figure 4b). Those detected in at least 25% of the blocks were then compared with HB data collected for the same colonies in order to identify proteins whose expression patterns correlated with the behavior (see Methods). Briefly, a Linear Mixed Effects model was used to estimate the effect of each predictor variable (e.g. geographic origin of the population and HB) on the protein expression level.

### Geographical origin affects protein expression

**Figure 5:**
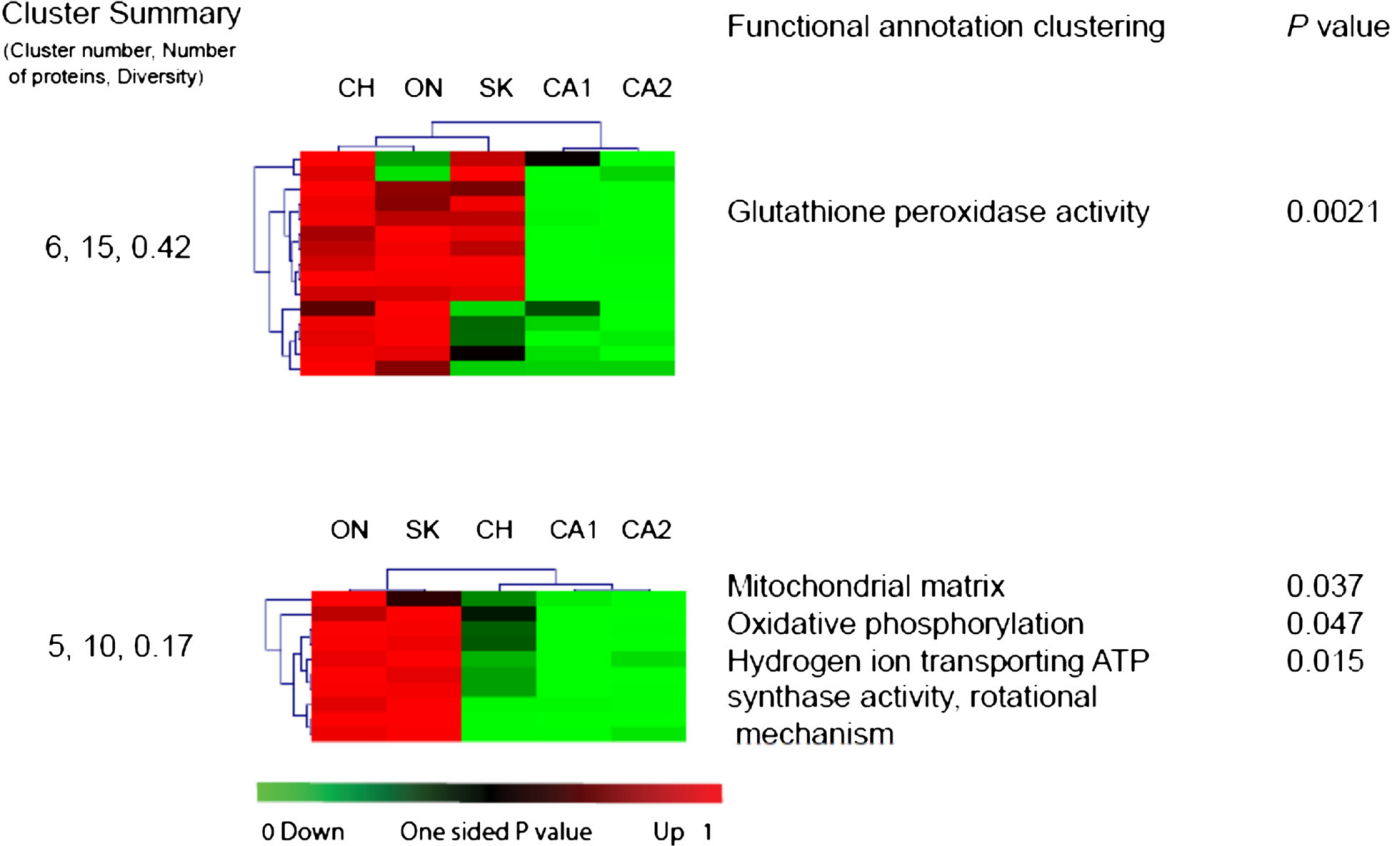
**Clustering and functional enrichment of proteins regulated by geographical origin.** Proteins regulated by geographical origin were clustered (SOTA) based upon statistical significance and relative abundance. Using orthologous proteins derived from *Drosophila* the function of bee proteins were predicted and each cluster was tested for the enrichment of gene ontology categories biological processes, cellular compartment and molecular function using DAVID (see Methods).

Given our previous observations [27] we first asked whether any of the protein expression profiles could be explained, at least in part, by the geographical origin of the colonies? Of the 476 proteins in at least 25% of the blocks, the profiles for thirty eight showed a statistically significant effect of geographical origin (Q < .05) and these were then subjected to gene enrichment analysis as described previously [27]. Hierarchical clustering of heat-maps enabled the visualization of two major protein clusters associated with specific biochemical functions (Figure 5, Additional file 1: Table S2). Enrichment analysis indicated that bees of Canadian and Californian origin are divergent in expression of proteins involved in mitochondrial respiration (e.g. ATP-synthase, Cytochrome C) and glutathione detoxification (Glutathione-S transferase S1, Peridoxin). The Chilean bees were divergent, sharing reduced expression of mitochondrial enzymes with Californian and increased expression of Glutathione detoxification proteins with Canadian varieties.

The effect of population origin on the relative abundance of proteins in the antenna supports our previous observation that historic latitudinal clines are detectable in the expression of metabolic proteins in imported bees [27]. The co-regulation of proteins driving oxidative metabolism with those of glutathione mediated detoxification is consistent with the management of oxidative stress associated with increased metabolic rates. In our analysis of population origin, no functional enrichment for neurological proteins was observed compared to the antenna proteome indicating that basic neurological function or structure was similar between the bees of different geographical origin.

### Proteins correlated with HB

**Figure 6:**
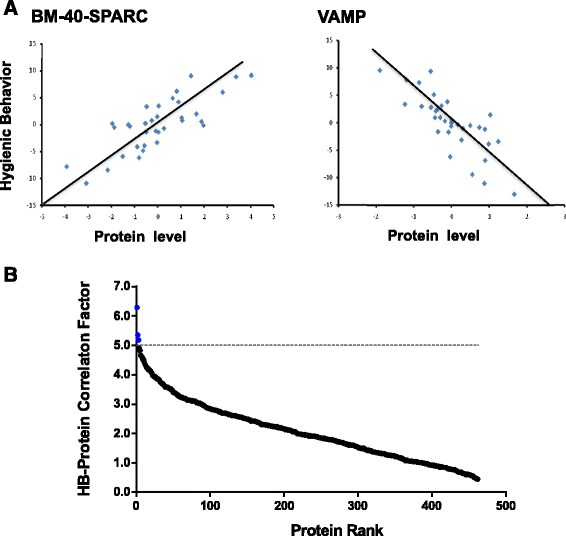
**Identification of proteins correlating with HB. A**. Partial regression plots of HB versus protein level. Results from BL-Y1 colonies showed two proteins with a strong correlation with HB. The P-values for BM-40-SPARC/ GI: 66512788 and VAMP/ GI:48138819 were 0.00017 and 0.00024, respectively and the Q-values were 0.05562 for both proteins. **B**. Final HB correlation score of proteins from all datasets calculated as described in Methods. BM-40-SPARC, Odorant binding protein 18/ GI:110774625 and 26S protease regulatory subunit 6A/ GI:48101907 showed an HB correlation factor >5.

We then studied whether variations in protein expression were associated with behavioral differences, and if they could be predictive markers of social immunity, focusing on HB (Additional file 2: Table S3). Among the Y1 colonies at the Alberta breeding site, the expression patterns of GI:48138819 (XP_393426.1) and GI: 66512788 (XP_392466.2) showed a strikingly strong correlation with HB (P < .0005, Q < .1, Figure 6). Proteins that are intrinsically involved with a behavior should be correlated with that behavior independent of where the measurements are taken and as such we examined which proteins were consistently correlated with HB at both breeding sites. The proteins strongly correlated with HB (P < .05) at both locations were Odorant binding protein 16/GI:94158709, Calcyclin Binding Protein/GI: 66564402, 3-ketoacyl-CoA thiolase/GI: 48097100, and VAMP (vesicle-associated membrane protein)/GI:48138819, found previously. The other protein observed initially, GI: 66512788, had too many missing data at the GF breeding site to be considered. We then extended this approach to cover Y1, Y2 and Y3, particularly to incorporate heritability information obtained from the Y3 BC partial diallel cross (Figure 2). Over the six datasets (Figure 2), proteins were ranked based on a HB-correlation factor average of all datasets plus a heritability factor (see Methods). Of the top ranked proteins, we also required that they were quantitated in at least four of the six datasets. BM-40-SPARC, Odorant binding protein 18/GI: 110774625 and 26S protease regulatory subunit 6A/GI: 48101907 showed the highest HB correlation factors when considering all datasets. Compiling these approaches, the seven proteins that emerged (Table 1) with expression patterns were most closely correlated with HB, are thus putatively useful for marker assisted selective breeding of HB and also suggest some testable hypotheses regarding the mechanism(s) underlying HB.

**Table 1:** **Proteins most highly correlated with HB**

**Accession #**	**Protein markers**	**Direction**	**General function**
GI: 110774625	Odorant binding protein 18	↑	Ligand binding
GI: 94158709	Odorant binding protein 16	↑	Ligand binding
GI: 48138819	VAMP (vesicle-associated membrane protein)	↓	Nerve signal transduction
GI: 66512788	BM-40-SPARC (Secreted protein acidic and rich in cysteine Ca binding)	↑	Nerve signal transduction
GI: 66564402	Calcyclin Binding Protein	↑	Signal down-regulation
GI: 48097100	3-ketoacyl-CoA thiolase, mitochondrial-like	↑	Ligand degradation
GI: 48101907	26S protease regulatory subunit 6A	↑	Signal down-regulation via protein degradation

### Exploring potential ligands of OBPs linked to HB

Among the seven HB-associated proteins (Table 1) two were odorant-binding proteins (OBPs), suggesting that hygienic bees may be sensing something emitted from diseased or dying larvae. The natural ligands are unknown for most bee OBPs, however, so we purified recombinant versions of the two high correlated OBPs detected here (OBP16, OBP18) and screened their affinity towards several metabolites in competitive binding assays. As reference, we chose OBP21, which is a ‘C-minus’ OBP, like OBP16 and OBP18, containing only four of the six conserved cysteines present in classic OBPs [28]. We had also characterized OBP21 in terms of ligand-binding affinities [29].

Affinities of twenty-nine ligands to the three proteins were evaluated in competitive binding assays, using N-phenyl-naphthylamine (1-NPN) as a fluorescent reporter and measuring the fluorescence decrease produced by the addition of the ligand in a concentration dependent fashion. Actual dissociation constants were calculated from the concentrations of each ligand halving the initial fluorescence of the complex ([IC]_50_) as described in Methods.

**Figure 7:**
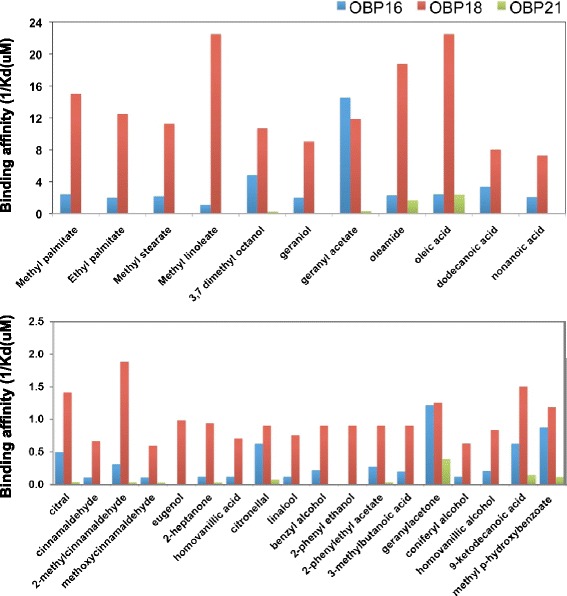
**Potential ligands of OBPs.** Twenty nine potential ligands were tested in a competitive binding assay for their binding to OBP16, OBP18 and OBP21. Each purified recombinant protein was mixed with the fluorescent probe N-phenyl-1-naphthylamine (1-NPN), both at the concentration of 2 μM in Tris buffer. Each mixture was titrated with a ligand and the displacement of the fluorescent probe from the complex was used to evaluate the relative dissociation constants, as described in the Methods section. Compounds with higher affinities are shown in the top panel, while compound with lower affinity are shown in the lower panel. OBP16 and OBP18 showed higher affinities for most compounds than the previously characterized OBP21. Of particular interest is the high binding affinity of OBP18 to oleic acid.

Potential ligands were selected from among the terpenoids commonly occurring in the scent of flowers, fatty acids and their esters, components of the queen mandibular pheromone and the brood pheromone and other volatiles potentially linked to hygienic behavior [30,31]. Figure 7 shows the binding affinity of the twenty-nine compounds tested; the upper panel reports the data for the strongest ligands, whose affinities are about one order of magnitude higher than the ligands reported in the lower panel. Interestingly, OBP16 and OBP18 bind to nearly all compounds tested with higher affinity than OBP21.

**Figure 8:**
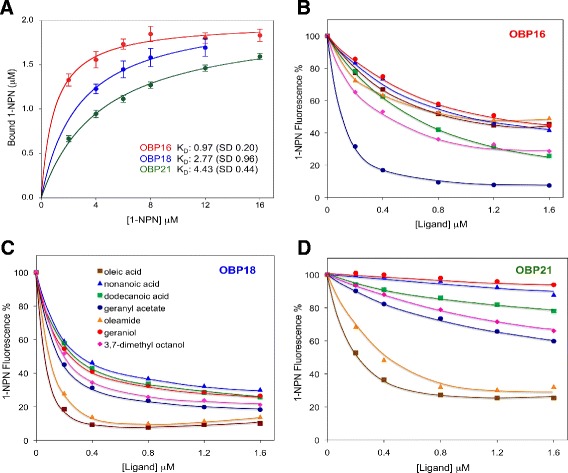
**Differential ligand binding to OBPs. A**. Binding curve of OBPs to 1-NPN, showing dissociation constants in the range of 1 to 5 μM which allowed for an accurate evaluation of the affinities towards other ligands in competitive binding assays. Purified recombinant proteins at the concentration of 2 μM in Tris buffer were titrated with increasing amounts of the fluorescent probe. **B**, **C** and **D**: Competition curves for the seven ligands with the highest affinities for OBP16, OBP18 and OBP21 respectively using the procedure described in Figure 7 and in the Methods section.

The three OBPs bind N-phenyl-1-naphthylamine (1-NPN) with dissociation constants in the range of 1 to 5 μM (Figure 8), similar to most other insect OBPs [29,32], thus allowing accurate evaluation of the affinities towards other ligands in competitive binding assays. All IC50 and K_d_ values are listed in Additional file 3: Table S4, while the dissociation curves for the strongest ligands are shown in Figure 7. In general, all three OBPs tended to prefer fatty acids and their ester and amide derivatives, as well as three structurally similar terpenoids: 3,7-dimethyloctanol, geraniol and geranyl acetate. OBP16 and OBP18 can both accept linear and branched molecules while OBP21 seems to have greater specificity to linear chains of 18 carbon atoms. OBP18 showed the highest affinities for several compounds including oleic acid which is released by decaying insect corpses [33].

## Discussion

Honey bees are an essential component of human agriculture but are under ever-increasing threat from parasites and infectious diseases. While acaricides, fungicides and antibiotics have been useful for controlling many bee pathogens and pests, resistance to these products is spreading and there is substantial public pressure to move away from such chemical controls. In-hive chemical treatments can leave residues in honey and other hive products, and in the environment. In addition, they can have sublethal effects on bees including potential effects on their immune system [34]. Selection of bees with higher disease resistance may reduce the need of chemical treatments and some disease-resistance traits have been identified in bees. Though honey bees can be selectively bred, the traits for disease resistance are, however, difficult to test and only a few, highly specialized groups, usually at universities or in technical transfer teams are able to use them (e.g., hygienic behavior, grooming, *Varroa*-sensitive hygiene) [35]. If molecular markers correlated with disease resistant traits were known, bee breeders could potentially utilize these in a more effective selective breeding program by employing molecular diagnostics in place of field behavioral assays. One typically thinks of such markers at the level of DNA (e.g., quantitative trait loci, single-nucleotide polymorphisms) but there has been little progress towards identifying any loci linked to disease-resistant behaviors in bees [36,37]. Nevertheless, protein expression levels could be marker of disease resistance. Indeed, the proteins identified would likely be more closely linked to the mechanism behind the behavior than a DNA feature.

### Protein biomarkers and prospects for marker-assisted selective breeding in bees

HB is an economically beneficial, heritable trait that enables bees to co-exist with pathogens and as such we have undertaken an extensive and exhaustive search for proteins whose expression levels are highly correlated with HB. We chose to focus on expression levels within the antennae of nurse bees because it is this particular behavioral ontogenic stage that performs the HB in the colony and one of the likely mechanisms for HB is a heightened ability to sense dead, dying or diseased larvae [38], which would likely involve antennae. Despite these experiments being performed in the field with genetically diverse, outbred populations, the results provided a strikingly clear confirmation of our hypothesis, that there is indeed a protein expression pattern unique to HB.

The discovery of biomarkers specific to HB allows for the possibility to develop prognostic assays that could be used to select parent colonies in a marker-assisted selection breeding program. We envision that the HB markers reported here will be useful to facilitate selective breeding efforts. Future work will aim to validate and apply these biomarkers in a marker-assisted selective breeding program with the goal of enriching honey bee populations for this social immunity trait. Beyond this potentially practical application, the proteins whose expression levels were most highly correlated with HB are all obviously linked to various aspects of chemosensory processes, suggesting several very interesting and testable hypotheses regarding the mechanism(s) underlying HB.

### Sensing the signal for HB

Insects rely on chemical communication to monitor the environment and exchange information between conspecifics. Social insects, in particular have developed a highly sophisticated chemical language, enabling members of the colony to perform different tasks. The chemoreception system of insects is mediated by olfactory receptors, located on the membrane of sensory neurons, and by soluble proteins present at high concentration in the lymph of chemosensilla [32,39]. These proteins belong to two major classes, OBPs (odorant-binding proteins) and CSPs (chemosensory proteins), in both cases small polypeptides folded into α-helical domains, arranged in two different unique motifs [40]. Although the specific action of OBPs and CSPs is not yet clear, certainly airborne molecules, as those associated with diseased or dead bees, upon entering the antenna interact with these soluble proteins and are tightly bound to be carried through the aqueous environment of the sensillar lymph to membrane receptors [39]. Thus, if hygiene in bees is due to an enhanced sensitivity towards specific signals originated in the affected brood, OBPs would probably be effecting this and OBP16 (GI: 94158709) and OBP18 (GI: 110774625) are the most likely candidates, based on the data presented here. Neither OBP16 nor OBP18 are exclusive to antennae, although their tissue expression patterns are consistent with a sensory molecule. OBP16 is expressed exclusively in peripheral tissues, including antennae, and is found in all castes, although it is most highly expressed in workers [29,41,42]. OBP18 is also largely in peripheral tissues but is also concentrated in the nerve cord of the female castes [41]. Clearly this is where OBPs should be, but what is it that they are detecting when they are expressed? The natural ligands of most bee OBPs, including these two, are unknown but our investigations here with recombinant proteins suggest that both OBP16 and OBP18 prefer branched and linear fatty acids. This general class of molecules includes many bee pheromones [43] so is consistent with a role for them in HB. Of particular interest is the high affinity of OBP18 to oleic acid because it is released by decomposing insects [33] and may be a strong mediator for social immunity in *Apis mellifera* and other eusocial species. Further demonstration of the precise ligand(s) they are detecting would require electroantennogram tests of selected compounds in bees that have had one OBP or the other knocked down by siRNA.

### Transmitting the signal for HB

Two proteins involved in inter-nerve communication were the most tightly linked to HB: vesicle-associated membrane protein (VAMP, GI: 48138819) and secreted protein acidic and rich in cysteine Ca binding (BM-40-SPARC, GI: 66512788). As any animal behavior requires peripheral, if not also central nervous system activity, it seems reasonable that a heightened behavior could result from up- or down-regulation of proteins required in signal propagation. VAMP is a well-known SNARE protein required for fusing synaptic vesicles at the synaptic cleft to release neurotransmitters [44] and as such our observation that it is inversely correlated with HB would suggest that it is particularly important in an inhibitory synapse and that its expression needs to be suppressed for neurons involved in HB to fire properly. BM-40-SPARC (a.k.a. testican in mammals) is a proteoglycan whose function is not understood but its transcript is up-regulated in nurse bees [45], which are the bees that performs HB, and it is implicated in brain development in mammals [46].

### Degrading the signal for HB and down-regulating the response

An important aspect of a response to any signal, particularly the depolarization involved in triggering a nerve response, is the termination of the signal to allow the system to be reset so that it may respond again. The remaining three proteins correlated with HB appear to fall within this this category:

3-ketoacyl-CoA thiolase (EC2.3.1.16, GI: 48097100) is involved in beta-oxidation and catalyzes the conversion of acyl-CoA and acetyl-CoA to CoA by itself and 3-oxoacyl-CoA. The isoform found here is likely the mitochondrial version, which would suggest that its key role is in energy production. This could indicate a specific energy requirement for hygienic behavior but this enzyme also happens to degrade the same class of molecules that acts as ligands for OBP16 and OBP18 so it is tantalizing to speculate that it may also act to shut down the signal for HB.

Calcyclin binding protein (GI: 66564402) is involved in targeting specific signalling proteins for degradation in other organisms, implying that it may be involved in degrading components of the signalling involved in HB, perhaps the OBPs themselves. Calcyclin binding protein is known as Siah-interacting protein in humans but it is not clear that its interaction with calcyclin there has any functional relevance. Structural analysis of Siah and Siah-interacting/calcyclin-binding protein indicates that calcyclin-binding protein is a component of an E3 ligase complex and that it is required to recruit an E2-substrate complex for the final step of ubiquitin transfer [47]. It has been most-studied in the context of signalling oncogenes so it is attractive to speculate that it may also be involved in degrading proteins involved in the signalling behind HB and thereby down-regulating the signal.

26S protease regulatory subunit 6A (GI: 48101907) appears to be a multi-functional protein and it is not clear which of its roles might be relevant in HB. It is a component of the 26S proteasome [48], which degrades ubiquitylated proteins, therefore Tat-binding protein might somehow help to turn over other proteins directly involved in HB, such as those above. Given that calcyclin-binding protein is also involved in ubiquitin-mediated protein degradation, it seems most likely that it is in this capacity that Tat-binding protein is involved in HB too. However, it is also a transcriptional co-activator [49] of hormone receptors (a mammalian functional equivalent of OBPs) so it could conceivably be regulating the expression of other proteins involved in HB, such as OBP16 and OBP18.

## Conclusions

Since protein expression can be influenced by environment and the technology for measuring proteins has lagged behind tools for measuring nucleic acids, protein markers has often been ignored in favor of QTLs or SNPs for marker-assisted selection for breeding purposes. Nevertheless, the link between QTLs or SNPs and phenotype can also be influenced by environment in most cases and so there is no intrinsic reason for proteins not to be investigated as biomarkers. To this end, we have shown that the expression levels of a selected set of proteins are heritably associated with an important social immunity trait in honey bees, hygienic behavior. Our data suggest that bees expressing this trait are better able to detect and respond to a chemical signal emitted by diseased or dying larvae, stimulating them to remove the potential threat from the colony environment. The chemical signal responsible for this remains to be identified but the proteins described here should make suitable biomarkers to guide selective breeding for hygienic behavior.

## Methods

### Establishing bee populations, HB testing, breeding and sample collection

The collection of honey bee samples, field testing and breeding was performed at two breeding locations in Western Canada, one near Grand Forks, BC (49°N, 118°W), the other at the Research Farm of Agriculture and Agri-Food Canada in Beaverlodge, AB (55°N, 119°W). An initial experiment was performed in BC as part of the BC Bee Breeders Association Queen Testing Project to confirm that hygienic behavior could be selectively bred for in our apiaries. In this experiment, selection to enrich for HB was based on field testing using the freeze-killed brood assay explained below. A second experiment was performed both in BC and AB with the aim to correlate proteome profiles with field test results in search for biomarkers of HB. For this breeding and proteomic experiment, the year 1 (Y1) colonies in BC included stock spanning a range of HB and *Varroa* resistance, including local and broader Canadian stock selected for mite resistance or HB, as well as descendants of a close-mated population at the University of Minnesota inbred for hygienic behavior [20,50] and VSH lines [51,52]. The starting colonies in AB consisted of eight populations described previously [27] of which five were sampled for proteomic analysis; these originated from Ontario (ON), California 1 (CA1), California 2 (CA2), Chile (Ch), and Saskatchewan (SK). Instrumental insemination (ii) was used for all breeding, except for the Y2 breeding in BC. Instrumental insemination of virgin queens from high- and low-scoring colonies followed a partial diallel cross design [53] which created high and low scoring colonies, as well as hybrids, intended to facilitate the identification protein expression patterns unique to HB. In Y3, we also performed ii of virgin queens in BC to evaluate the heritability of the protein markers identified. All inseminated or closed mated queens were introduced into new colonies. After the queens started laying, colonies were allowed to develop for at least six weeks to allow worker populations to turn over before they were tested for HB and antennae were collected for proteomic analysis. Colonies were assessed for HB using the freeze-killed brood method [14], where the proportion of sealed cells that nurse bees uncap (uncapped, U) and remove dead pupae from (removed, R) is counted at 24 and 48 h using two separate tests performed one week apart on each colony. For proteomic analysis, antennae were cut from adult workers sampled from brood frames (three pools of ten bees from each colony). Invertebrate research (except on cephalopods) does not require ethics certification at our institution.

### Reagents

All chemicals used were of analytical grade or better and all solvents were of HPLC-grade or better; all, with the exceptions specified below, were obtained from ThermoFisher-Scientific (St. Waltham, MA, USA). Chemicals for protein expression and purification and for binding assays were purchased from Sigma-Aldrich and were of reagent grade, with the exception of selected compounds used in binding assays, that were prepared using conventional synthetic routes. Selected reagents were purchased from the following commercial sources: Endopeptidase Lys-C (Wako Chemicals, Osaka, Japan); porcine modified trypsin (Promega, Nepean, Ontario, Canada); loose ReproSil-Pur 120 C18-AQ 3 μm (Dr Maisch, Ammerbuch-Entringen, Germany); 96-well full skirt PCR plates (Axygen, Union City, CA, USA); fused silica capillary tubing (Polymicro, Phoenix, AZ, USA); protease inhibitor mixture (Roche Applied Science, Basel, Switzerland); NuPAGE Novex BisTris Gels (Invitrogen, Carlsbad, CA, USA). All cloning enzymes were from New England Biolabs. Oligonucleotides were custom synthesized at Eurofins MWG GmbH, Ebersberg, Germany.

### Matrix for sample analysis

The isotopic labelling strategy employed here is limited to triplexing so to enable a comparison of the protein expression profile for one colony to all others we employed a design similar to what we have done previously that maximized the statistical power of the experiment to detect effects in our parameters of interest [27]. We collected three replicate samples from each colony, grouped the samples in blocks of three, assigned one of the three isotopic labels to each sample, and assigned colonies to blocks in order to minimize the variance of the hygienic behavior variables. We constrained the experiment so no two colonies from the same population were in the same block and no two samples from the same colony were assigned the same isotopic label. This ensured the experimental design did not confound the hygienic behavior effect with the bee population or the isotopic label.

### Protein preparation for mass spectrometry

Bee antennae samples were washed three times with phosphate-buffered saline (PBS) and bead-homogenized in buffer (50 mM Tris-Cl, 150 mM NaCl, 1% NP-40, 1% DTT) for three 20 s bursts at 6.5 M/s, with 1 min rest on ice between each burst. Insoluble material was pelleted at 600 relative centrifugal force (RCF) and protein was precipitated from the supernatants using 800 μL of ethanol, 20 μL of 2.5 M sodium acetate (pH 5.5) and 2 μL of glycogen (10 mg/ml). The precipitation was allowed to proceed at room temperature for 90 min. After centrifugation at 16,000 r.c.f. for 15 min, the pellets were dried and solubilized in buffered urea (6 M urea, 2 M thiourea, 100 mM Tris-Cl at pH 8.0, 20 mM DTT). Any insoluble material was then removed by centrifugation at 16,000 r.c.f. for 15 min. Protein concentrations were measured by a micro Bradford assay using serial dilutions of bovine serum albumin to generate a standard curve. Protein samples were resolved on 1-D Nu-PAGE (Invitrogen) gels and visualized with Coomassie Safe Blue (Pierce) to check the protein stability and quantity. For each sample, 20 μg of protein was diluted to 1 μg/μl in urea buffer (6 M urea, 2 M thiourea, 100 mM Tris-Cl, pH 8.0) before digestion [54].

### Peptide clean-up and labelling

Ten micrograms of digested peptides were purified with STop And Go Extraction (STAGE) tips [55] and labelled via reductive dimethylation using formaldehyde isotopologues [56,57]. In each triplex block one sample received 10 μl of 200 mM CH_2_O (light) and 1 μl of 1 M NaBH_3_CN, one received 10 μl of 200 mM C^2^H_2_O (medium) and 1 μL of 1 M NaBH_3_CN and one received 10 μL of 200 mM ^13^C^2^H_2_O (heavy) and 1 μL of 1 M NaBH_3_CN. The labelling reaction was performed twice on each sample for 1 h each. The reactions were terminated by the addition of 20 μL of 3 M NH_4_Cl. Samples were adjusted to pH <3 by adding sample buffer (3% (w/v) acetonitrile, 1% (v/v) trifluoroacetic acid, 0.5% (v/v) acetic acid). Finally, 4 μg of each of the three differentially-labeled samples were combined and cleaned up again with a STAGE tip; two technical replicates were prepared for each block, with one to be analyzed on the LTQ-FT and the other on the LTQ-Orbitrap. Samples were stored on STAGE tips at 4°C as needed.

### Liquid chromatography-tandem mass spectrometry (LC-MS/MS)

Peptides were eluted from the STAGE tips using elution buffer (0.1% trifluoroacetic acid, 80% acetonitrile). Then they were dried and resuspended in sample buffer (1% trifluoroacetic acid, 3% acetonitrile, 0.5% acetic acid). LC-MS/MS was performed using an 1100 Series nanoflow high performance liquid chromatography system (Agilent Technologies) on-line coupled to a linear trapping quadrupole (LTQ)-Fourier transform (FT) or a LTQ-Orbitrap (ThermoFisher Scientific, Bremen, Germany). Peptide separation was performed by reversed phase chromatography using a 75 μm inner diameter fused silica emitter self-packed with 3 μm Reprosil-Pur C_18_-AQ resin (Dr. Maisch). Peptides were loaded in 4.8% (v/v) acetonitrile, 0.5% (v/v), acetic acid at 0.6 μL/min and then resolved at 200 nL/min for 75 min using a linear gradient of acetonitrile from 4.8% to 64% in 0.5% (v/v) acetic acid. Operating in data dependent mode, the LTQ-FT was set up to acquire full scan data in the FT detector over a mass range of 350–1600 m/z before performing FT selected ion monitoring (SIM) and MS/MS in the ion trap on the top 3 most intense multiply charged ions [58]. The LTQ-OrbitrapXL was set to acquire a full-range scan at 60,000 resolution from 350 to 1600 Th in the Orbitrap to simultaneously fragment the top five peptide ions in each cycle in the LTQ (minimum intensity 1000 counts). Parent ions were then excluded from MS/MS for the next 30 s. Singly-charged ions were excluded since in ESI mode peptides usually carry multiple charges. The Orbitrap was continuously recalibrated using the lock-mass function.

### Protein identification and quantification

Fragment spectra peak lists were created using DTASuperCharge [59] with default parameters. For each block of samples, the peak list generated from LTQ-FT was combined with the peak list from LTQ-Orbitrap before performing Mascot search (v2.2) against the Honey Bee, *A. mellifera* Amel_4.0 translation (forward plus reversed sequences) of the genome with additional entries for human keratins, porcine trypsin and LysC. Tryptic cleavage rules (R/K, except preceding a P) were specified with up to two missed cleavages allowed. Carbamidomethyl (C) was set as a fixed modification, Acetyl (Protein N-term), Deamidated (NQ), Oxidation (M), Dimethyl (K), Dimethyl (N-term), Dimethyl: 2H(4) (K), Dimethyl: 2H(4) (N-term), Dimethyl: 2H(6)13C(2) (K), Dimethyl: 2H(6)13C(2) (N-term) as variable modifications. Peptide tolerance was set to 10 ppm and MS/MS tolerance was 0.6 Da for the initial search. After recalibration of systematic mass errors the peptide mass accuracy is typically <2 ppm. The false discovery rate within each block was limited to 1%, estimated by counting the number of ‘hits’ against the reversed sequences. Across the whole experiment, however, the FDR approaches zero since no reversed hits survived the filter requiring that a protein had to be detected in at least one quarter of all blocks. All peptides with an IonsScore ≥25 were quantified using MSQuant (v1.5) [59]; after automated quantitation all files were manually edited to ensure consistent quantitation and the peak area ratios were exported for further analysis. An in-house script, finalList.pl (available here: http://www.chibi.ubc.ca/wp-content/uploads/2013/01/finalList.pl_.txt) for applying parsimony (Occam’s razor) to generate a non-redundant list of identified proteins from a large pool of independent experiments was adapted to simultaneously calculate average peptide ratios for each protein in each block. All raw data are available from the Honey Bee Peptide Atlas (http://www.peptideatlas.org/builds/honeybee/) and ProteomeXchange (identifier PXD001616) while all the proteins identified and their quantitative ratios can be found in Additional file 4: Table S1.

### Statistical analysis and marker selection

Identification of proteins whose expression patterns correlated with population of behavioral data was performed as described previously [27]. Briefly, logarithms of intensities were normalized by first subtracting the average of the three measurements in each block (for each protein independently) and then centering and standardizing within each label (across proteins) by the median and median absolute deviation. For each protein, a Linear Mixed Effects model was used to estimate the effect of each predictor variable, either population or hygienic behavior, on the protein expression level, adjusting for block and label factors. In the case of the BL-Y1 dataset, analysis of the effect of hygienic behavior was done adjusting for population of origin. For the predictor variables, an estimated effect, standard error and P-value were computed for each protein response. FDRs (Q-values) were computed for the set of P-values of a given predictor over all protein response variables to adjust for multiple comparisons. All calculations were performed in the R statistical language. In addition to selecting markers with low Q values from the BL-Y1 dataset, we used the Y1 colonies from the two different apiaries and selected proteins that had P < .05 across all field parameters in both datasets. After completing the proteomic analysis of all Y1, Y2 and Y3 datasets, we further selected proteins by ordering them based on an overall HB correlation factor The HB correlation was computed by adding a heritability factor to an average of the HB factors computed from each dataset. The HB factor in each dataset was calculated by combining a biological and statistical factor as detailed in the Additional 5. The heritability factor for each protein, was based on a regression of the protein level observed in the F1 daughters on the observed level in the paternal (Sir) and maternal (Dam) parent colonies (see Additional 5 for more details).

### Expression clustering and gene ontology enrichment

SOTA (self-organizing tree algorithm) clustering was used to determine one side probability metrics for all thirty-eight population-significant (Q ≤ .05) proteins across all honey bee populations. Using MultiExperiment Viewer, six hard clusters were generated and hierarchical dendrograms for population and proteins were constructed using Euclidean distances [60]. For each cluster, gene ontology (GO) enrichment analysis was performed based on the *Drosophila* orthologs to the complete protein sequence of the bee proteins identified. DAVID (Database for Annotation, Visualization, and Integrated Discovery) [61,62] was used to calculate enrichments between protein lists of interest using the entire identified antenna proteome characterized here (470 proteins) as background.

### Expression of recombinant proteins and binding assays

#### RNA extraction and cDNA synthesis

Total RNA was extracted using TRI® Reagent (Sigma), following the manufacturer’s protocol. cDNA was prepared from total RNA by reverse transcription, using 200 units of SuperScript™ III Reverse Transcriptase (Invitrogen) and 0.5 mg of an oligo-dT primer in a 50 μL reaction volume. The mixture also contained 0.5 mM of each dNTP (GE-Healthcare), 75 mM KCl, 3 mM MgCl_2_, 10 mM DTT and 0.1 mg/ml BSA in 50 mM Tris–HCl, pH 8.3. The reaction mixture was incubated at 50°C for 60 min and the product was directly used for PCR amplification or stored at −20°C.

#### Polymerase chain reaction

Aliquots of 1 μL of crude cDNA were amplified in a Bio-Rad Gene Cycler thermocycler, using 2.5 units of *Thermus aquaticus* DNA polymerase (GE-Healthcare), 1 mM of each dNTP (GE-Healthcare), 1 μM of each PCR primer, 50 mM KCl, 2.5 mM MgCl_2_ and 0.1 mg/ml BSA in 10 mM Tris–HCl, pH 8.3, containing 0.1% v/v Triton X-100. At the 5’ end, we used specific primers corresponding to the sequence encoding the first five amino acids of the mature protein. The primers also contained an NdeI restriction site, for ligation into the expression vector and providing at the same time the ATG codon for an additional methionine in position 1. At the 3’ end specific primers were used, encoding the last six amino acids, followed by a stop codon and an EcoRI restriction site for ligation into the expression vector. Therefore, we used the following primers for the each protein (enzyme restriction sites are italicized):

fw*Amel*OBP16: 5’- GAGGAATAA*CATATG*ACACATGAGGAATT -3’

rv*Amel*OBP16: 5’- *GAATTC*TTAGGAATTTAATATATCAGT -3’

fw*Amel*OBP18: 5’- GAGGAATAA*CATATG*ACACTTGAAGAATT -3’

rv*Amel*OBP18: 5’- *GAATTC*TTAGCCACTTAACATTTCTTT -3’

After a first denaturation step at 95°C for 5 min, we performed 35 amplification cycles (1 min at 95°C, 30 s at 50°C, 1 min at 72°C) followed by a final step of 7 min at 72°C. We obtained amplification products of 300–400 bp, in agreement with the expected sizes.

#### Cloning and sequencing

The crude PCR products were ligated into a pGEM (Promega) vector without further purification, using a 1:5 (plasmid:insert) molar ratio and incubating the mixture overnight, at room temperature. After transformation of *E. coli* XL-1 Blue competent cells with the ligation products, positive colonies were selected by PCR using the plasmid’s primers SP6 and T7 and grown in LB/ampicillin medium. DNA was extracted using the Plasmid MiniPrep Kit (Euroclone) and custom sequenced at Eurofins MWG (Martinsried, Germany).

#### Cloning in expression vectors

pGEM plasmids containing the appropriate sequences were digested with Nde I and Eco RI restriction enzymes for 2 h at 37°C and the digestion products were separated on agarose gel. The obtained fragments were purified from gel using QIAEX II Extraction kit (Qiagen) and ligated into the expression vector pET5b (Novagen, Darmstadt, Germany), previously linearized with the same enzymes. The resulting plasmids were sequenced and shown to encode the mature proteins.

#### Preparation of the proteins

For expression of recombinant proteins, each pET-5b vector containing the appropriate odorant-binding protein (OBP) sequence was used to transform BL21(DE3)pLysS and BL21(DE3)Rosetta-gami *E. coli* cells, for OBP18 and OBP16 respectively. Protein expression was induced by addition of IPTG to a final concentration of 0.4 mM when the culture had reached a value of O.D._600_ = 0.8. Cells were grown for further 2 h at 37°C, in the case of OBP18, while they were grown overnight at 30°C for OBP16 expression. They were then harvested by centrifugation and sonicated. After centrifugation, OBP16 was soluble while OBP18 was present as inclusion bodies. To solubilize it, the pellet from 1 L of culture was dissolved in 10 mL of 8 M urea, 1 mM DTT in 50 mM Tris buffer, pH 7.4, then diluted to 100 mL with Tris buffer and dialysed three times against Tris buffer.

Purification of the proteins was accomplished by combinations of chromatographic steps anion-exchange resins, such as DE-52 (Whatman), QFF or Mono-Q (GE-Healthcare), followed by gel filtration on Sephacryl-100 or Superose-12 (GE-Healthcare) along with standard protocols previously adopted for other odorant-binding proteins [63,64]. The electrophoretic analysis of crude bacterial pellets and representative fractions from the last purification steps for OBP16 and OBP18 are shown in Additional file 6: Figure S1.

#### Fluorescence measurements

Emission fluorescence spectra were recorded on a Jasco FP-750 instrument at 25°C in a right angle configuration, with a 1 cm light path quartz cuvette and 5 nm slits for both excitation and emission. The protein was dissolved in 50 mM Tris–HCl buffer, pH 7.4, while ligands were added as 1 mM stock solutions in methanol.

#### Fluorescence binding assays

To measure the affinity of the fluorescent ligand 1-NPN (N-phenyl-1-naphthylamine) to each protein, a 2 μM solution of the protein in 50 mM Tris–HCl, pH 7.4, was titrated with aliquots of 1 μM ligand in methanol to final concentrations of 2–16 μM. The probe was excited at 337 nm and emission spectra were recorded between 380 and 450 nm. The affinity of other ligands was measured in competitive binding assays, using 1-NPN as the fluorescent reporter at 2 μM concentration and 2–16 μM concentrations of each competitor.

To avoid the artefact provided by the strong fluorescent signals observed in the presence of ligands capable of forming micelles, such as long-chain fatty acids, we used 0.2-0.6 μM concentrations of each competitor. In fact, when this happens, the probe can bind inside the hydrophobic core of the micelle, emitting a signal similar to that produced in the binding pocket of a protein.

For determining binding constants, the intensity values corresponding to the maximum of fluorescence emission were plotted against free ligand concentrations. Bound ligand was evaluated from the values of fluorescence intensity assuming that the protein was 100% active, with a stoichiometry of 1:1 protein:ligand at saturation. The curves were linearized using Scatchard plots. Dissociation constants of the competitors were calculated from the corresponding IC_50_ values (concentrations of ligands halving the initial fluorescence value of 1-NPN), using the equation: K_D_ = [IC_50_]/1 + [1-NPN]/K_1-NPN_, [1-NPN] being the free concentration of 1-NPN and K_1-NPN_ being the dissociation constant of the complex Protein/1-NPN.

### Availability of supporting data

All mass spectrometry raw data used here is available in either Peptide Atlas (http://www.peptideatlas.org/builds/honeybee/) or the ProteomeXchange Consortium [65] via the PRIDE partner repository with the dataset identifier PXD001616”.

## Additional files

Additional file 1: Table S2. Clustering and functional enrichment data. The statistical analysis for the correlation of proteins and the geographical origins of the colonies Protein annotations can be found in tab 1. Tab 2 is the output of cluster analysis using the self organizing maps (SOM) procedure. Tab 3 and 4 give the gene ontology terms found enriched in protein clusters. Tab 5 provides a description of the column headings used in this supplementary data set. Additional file 2: Table S3. Protein-HB correlation data. Each of the first six tabs represents one generation at one breeding site. Protein accession numbers and statistical values are listed. The last two tabs provide protein heritability. Additional file 3: Table S4. Ligand binding data. Measured IC50 and calculated dissociation constants (K_D_) for 29 ligands tested with three OBPs of the honeybee. Values are all expressed in μM. Additional file 4: Table S1. Protein Expression and HB data. Relative ratios for all proteins found in at least 25% of the colonies in one breeding site/generation. Each of the first six tabs represents one generation at one breeding site. Protein accession numbers are listed on the left and block/colony combinations are listed across the top. The last tab contains all HB data. Additional file 5:
**Biological and Statistical factor calculations.** The Biological factor is the magnitude of the effect scaled by the average standard deviation from all the effects while the statistical factor is the negative log of the P-value scaled so a factor of 1.0 corresponds to a P-value of 0.01 calculated as follows: Biological factor = ABS(value)/ Average(STDEV), where ABS: is the absolute value, STDEV is the standard deviation and STDEV = SE*SQRT(DF), where SE is the standard error, SQRT(DF) is the square root of the degrees of freedom; Statistical factor = −LOG_100_(P-value). The use of LOG100 resulted in positive numbers of the same magnitude as the ABS(values). This is equivalent as defining a unit of the statistical factor as the negative log of 0.01 with base 100, −LOG_100_ of 0.01 = 1. A heritability value for each protein was estimated by fitting a regression model that predicted the level observed in the F_1_ daughter by the level observed in the paternal (Sir) and maternal (Dam) parent colonies. This gave us estimated effects for the protein levels of Sir and Dam parents as they relate to predicting F_1_ daughter protein levels. We then assigned a value based on whether the effect between the F_1_ progeny and the parents was higher than the median. If this was true for the two F_0_ parents a value of 2 was assigned. If it was true for only one F_0_ parent or for none of them, a value of 1 or 0 was assigned, respectively. Additional file 6: Figure S1. OBP purification. SDS-PAGE analysis of selected fractions from the last purification step of recombinant OBP16 and OBP18. M: molecular weight markers: 66, 45, 29, 24, 20 and 14 kDa. ni: bacterial pellet before induction with IPTG, i: bacterial pellet after induction. QFF: fractions containing purified OBP16 as eluted from strong anion exchange column QFF (GE-healthcare). S-100: fractions containing purified OBP18 as eluted from gel filtration column Sepharose-100 (Ge-Healthcare).
