# Supplementary material for: A search for protein biomarkers links olfactory signal transduction to social immunity
Source: BMC Genomics. 2015 Feb 8;16(1):63. doi: 10.1186/s12864-014-1193-6 (PMC4342888; doi:10.1186/s12864-014-1193-6)
Supplement: Additional file 5: — Biological and Statistical factor calculations. The Biological factor is the magnitude of the effect scaled by the average standard deviation from all the effects while the statistical factor is the negative log of the P-value scaled so a factor of 1.0 corresponds to a P-value of 0.01 calculated as follows: Biological factor = ABS(value)/ Average(STDEV), where ABS: is the absolute value, STDEV is the standard deviation and STDEV = SE*SQRT(DF), where SE is the standard error, SQRT(DF) is the square root of the degrees of freedom; Statistical factor = −LOG100(P-value). The use of LOG100 resulted in positive numbers of the same magnitude as the ABS(values). This is equivalent as defining a unit of the statistical factor as the negative log of 0.01 with base 100, −LOG100 of 0.01 = 1. A heritability value for each protein was estimated by fitting a regression model that predicted the level observed in the F1 daughter by the level observed in the paternal (Sir) and maternal (Dam) parent colonies. This gave us estimated effects for the protein levels of Sir and Dam parents as they relate to predicting F1 daughter protein levels. We then assigned a value based on whether the effect between the F1 progeny and the parents was higher than the median. If this was true for the two F0 parents a value of 2 was assigned. If it was true for only one F0 parent or for none of them, a value of 1 or 0 was assigned, respectively. [file 12864_2014_1193_MOESM5_ESM.doc]

**Supplemental Note**

Biological and Statistical factor calculations

The Biological factor is the magnitude of the effect scaled by the average standard deviation from all the effects while the statistical factor is the negative log of the P-value scaled so a factor of 1.0 corresponds to a P-value of 0.01 calculated as follows:

Biological factor = ABS(value)/ Average(STDEV), where ABS: is the absolute value, STDEV is the standard deviation and STDEV = SE*SQRT(DF), where SE is the standard error, SQRT(DF) is the square root of the degrees of freedom;

Statistical factor = -LOG_100_(P-value). The use of LOG100 resulted in positive numbers
of the same magnitude as the ABS(values). This is equivalent as defining a unit
of the statistical factor as the negative log of 0.01 with base 100, -LOG_100_ of
0.01 = 1.

A heritability value for each protein was estimated by fitting a regression model that predicted the level observed in the F_1_ daughter by the level observed in the paternal (Sir) and maternal (Dam) parent colonies. This gave us estimated effects for the protein levels of Sir and Dam parents as they relate to predicting F_1_ daughter protein levels. We then assigned a value based on whether the effect between the F_1_ progeny and the parents was higher than the median. If this was true for the two F_0_ parents a value of 2 was assigned. If it was true for only one F_0_ parent or for none of them, a value of 1 or 0 was assigned, respectively.
