# Supplementary figures and images for: A search for protein biomarkers links olfactory signal transduction to social immunity
Source: BMC Genomics. 2015 Feb 8;16(1):63. doi: 10.1186/s12864-014-1193-6 (PMC4342888; doi:10.1186/s12864-014-1193-6)

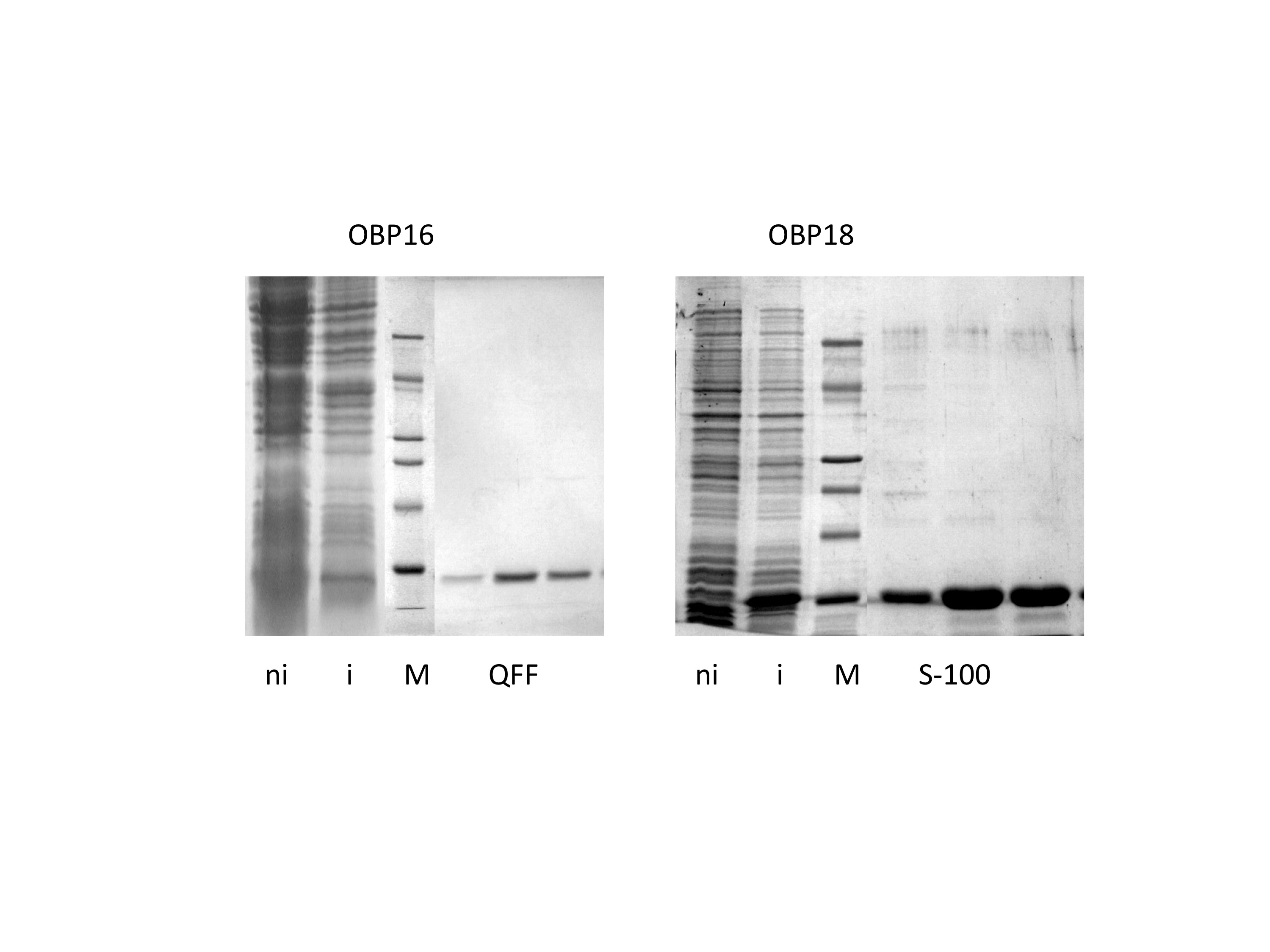

Supplement: Additional file 6: Figure S1. — OBP purification. SDS-PAGE analysis of selected fractions from the last purification step of recombinant OBP16 and OBP18. M: molecular weight markers: 66, 45, 29, 24, 20 and 14 kDa. ni: bacterial pellet before induction with IPTG, i: bacterial pellet after induction. QFF: fractions containing purified OBP16 as eluted from strong anion exchange column QFF (GE-healthcare). S-100: fractions containing purified OBP18 as eluted from gel filtration column Sepharose-100 (Ge-Healthcare). [file 12864_2014_1193_MOESM6_ESM.jpeg]
